# The ongoing risk of *Leishmania donovani* transmission in eastern Nepal: an entomological investigation during the elimination era

**DOI:** 10.1186/s13071-023-05986-9

**Published:** 2023-11-06

**Authors:** Lalita Roy, Kristien Cloots, Surendra Uranw, Keshav Rai, Narayan R. Bhattarai, Tom Smekens, Rik Hendrickx, Guy Caljon, Epco Hasker, Murari L. Das, Wim Van Bortel

**Affiliations:** 1 Tropical and Infectious Disease Centre, BP Koirala Institute of Health Sciences, Dharan, Nepal; 2 Department of Public Health, Institute of Tropical Medicine, Antwerp, Belgium; 3 Department of Internal Medicine, BP Koirala Institute of Health Sciences, Dharan, Nepal; 4 Department of Microbiology, BP Koirala Institute of Health Sciences, Dharan, Nepal; 5 Department of Biomedical Sciences, University of Antwerp, Antwerp, Antwerp, Belgium; 6 Department of Biomedical Sciences and Outbreak Research Team, Institute of Tropical Medicine, Antwerp, Belgium

**Keywords:** Visceral leishmaniasis, Vector, *Phlebotomus argentipes*, Seasonality, *Leishmania donovani* transmission, Infection rate, Host preference, Nepal

## Abstract

**Background:**

Visceral leishmaniasis (VL), a life-threatening neglected tropical disease, is targeted for elimination from Nepal by the year 2026. The national VL elimination program is still confronted with many challenges including the increasingly widespread distribution of the disease over the country, local resurgence and the questionable efficacy of the key vector control activities. In this study, we assessed the status and risk of *Leishmania donovani* transmission based on entomological indicators including seasonality, natural *Leishmania* infection rate and feeding behavior of vector sand flies, *Phlebotomus argentipes*, in three districts that had received disease control interventions in the past several years in the context of the disease elimination effort.

**Methods:**

We selected two epidemiologically contrasting settings in each survey district, one village with and one without reported VL cases in recent years. Adult sand flies were collected using CDC light traps and mouth aspirators in each village for 12 consecutive months from July 2017 to June 2018. *Leishmania* infection was assessed in gravid sand flies targeting the small-subunit ribosomal RNA gene of the parasite (SSU-rRNA) and further sequenced for species identification. A segment (~ 350 bp) of the vertebrate cytochrome b (*cytb*) gene was amplified from blood-fed *P. argentipes* from dwellings shared by both humans and cattle and sequenced to identify the preferred host.

**Results:**

Vector abundance varied among districts and village types and peaks were observed in June, July and September to November. The estimated *Leishmania* infection rate in vector sand flies was 2.2% (1.1%–3.7% at 95% credible interval) and 0.6% (0.2%–1.3% at 95% credible interval) in VL and non-VL villages respectively. The common source of blood meal was humans in both VL (52.7%) and non-VL (74.2%) villages followed by cattle.

**Conclusions:**

Our findings highlight the risk of ongoing *L. donovani* transmission not only in villages with VL cases but also in villages not reporting the presence of the disease over the past several years within the districts having disease elimination efforts, emphasize the remaining threats of VL re-emergence and inform the national program for critical evaluation of disease elimination strategies in Nepal.

**Graphical Abstract﻿:**

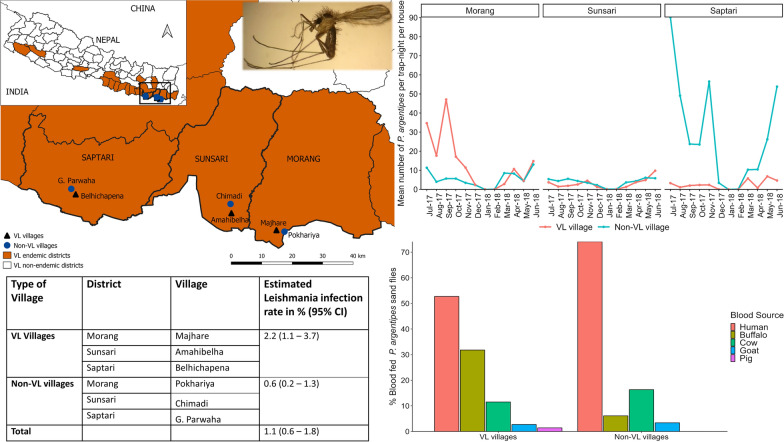

**Supplementary Information:**

The online version contains supplementary material available at 10.1186/s13071-023-05986-9.

## Background

Visceral leishmaniasis (VL), also known as kala-azar in the Indian subcontinent (ISC), is the most life-threatening form of leishmaniasis, with an estimated 50,000 to 90,000 new cases occurring worldwide every year [1]. Although India, Nepal and Bangladesh used to carry two-thirds of the global VL burden in 2004, this fraction was reduced to only 18% in 2020 [2] after a regional elimination initiative was launched in 2005. This initiative targeted the elimination of the disease from the ISC by 2015 [3], a deadline later extended to 2026 [4]. The elimination target was to reduce the annual incidence below one case per 10,000 population at the implementation unit level (district level in Nepal and sub-district level in India and Bangladesh) in all endemic areas, a target which is assumed to no longer form a public health concern [5]. VL was envisaged for elimination from the ISC because of some favorable factors like humans being the only known host or reservoir, *Phlebotomus argentipes* sand fly as the only vector transmitting the parasite *Leishmania donovani*, availability of chemical-based vector control tools, rapid disease diagnostic tools and complete treatment of the disease. Accordingly, the key strategies adopted in the region and in Nepal for disease elimination are early diagnosis and complete treatment, integrated vector management with main focus on indoor residual spraying (IRS) and effective disease and vector surveillance [6].

By 2013, the VL elimination target was achieved in all 12 officially endemic districts (districts where a full transmission cycle and therefore local transmission has been formally established, warranting the presence of the national VL elimination program) mostly situated in the central and eastern plain lowland areas (also called as “terai”) of Nepal. Nonetheless, 10 years after this first achievement, Nepal has still not been able to declare the elimination of VL as a public health problem. Several districts have breached the elimination target again since 2013, with two districts above the threshold in 2022. Also, the national VL elimination program added six districts in 2016 and five more in 2021 to the endemic district’s list, after the local transmission was epidemiologically and entomologically verified [2, 6, 7]. These newly endemic districts include hilly and mountainous areas from the eastern, central and western parts of the country. At present, 42 districts are listed as endemic, 30 districts as endemic doubtful (reports of at least one locally acquired VL case in the last 10 years and full cycle of transmission is yet to be verified) and only five districts as non-endemic (no locally acquired VL cases in the last 10 years) out of 77 total districts in Nepal (unpublished data).

The continued presence of sporadic VL cases even in the districts below the elimination threshold in the historical endemic areas sustains the risk of new outbreaks, local resurgence and transmission to neighboring unaffected areas. In this context, the elimination strategies need critical evaluation, realignment and reconsideration of relevance in the current phase of sustaining the elimination target and preventing re-emergence of disease transmission [5, 8]. One of the key strategies, i.e. vector control with synthetic pyrethroid-based IRS in human and animal shelters to reduce *L. donovani* transmission [9, 10], is vulnerable to poor performance because of logistic constraints, irregular operational practices and limited updated knowledge on vector bionomics [11–13]. Vector abundance and seasonality in an area are often related to the extent of vector-human contact and *Leishmania* transmission [14], and up-to-date information facilitates determining the appropriate spray timing for effective IRS implementation. Furthermore, even in the absence of reported VL cases, transmission can still be ongoing, as the vast majority of infections remain asymptomatic in humans, and clinical cases can be missed or can migrate elsewhere before diagnosis. Surveillance of the prevalence of *Leishmania* infection in vector sand flies can help to understand to what extent the parasite is still circulating in a certain area and provide crucial information for the prediction of the risk and expansion of leishmaniasis [15–18]. The role of a sand fly species as a vector is invariably associated with the intensity of host-vector contact for blood feeding; therefore, it is undoubtedly important to identify and quantify the source of blood meals in wild-caught vector sand flies to determine the risk of *Leishmania* transmission in the human population [19, 20].

Several studies investigated sand fly seasonality, prevalence of *Leishmania* in sand flies and host preferences in the early years of the elimination initiative, mostly to assess the *Leishmania* transmission dynamics and to check the efficacy of various vector control tools in Nepal [13, 17, 21–23]. As considerable changes in the epidemiological situation have been experienced in Nepal since then, updated information on these entomological indicators is sought to guide the VL elimination efforts for sustainable elimination of the disease in the country.

In this study, we aimed to assess the status and risk of *L. donovani* transmission in longstanding endemic districts by collecting data on seasonality, natural *L. donovani* infection rate and host preference of vector sand flies from two epidemiologically contrasting areas; villages with VL cases being reported in recent years and neighboring villages within the same endemic districts but without any such cases reported during the last 10 years in the eastern part of Nepal.

## Methods

### Study area

**Fig. 1:**
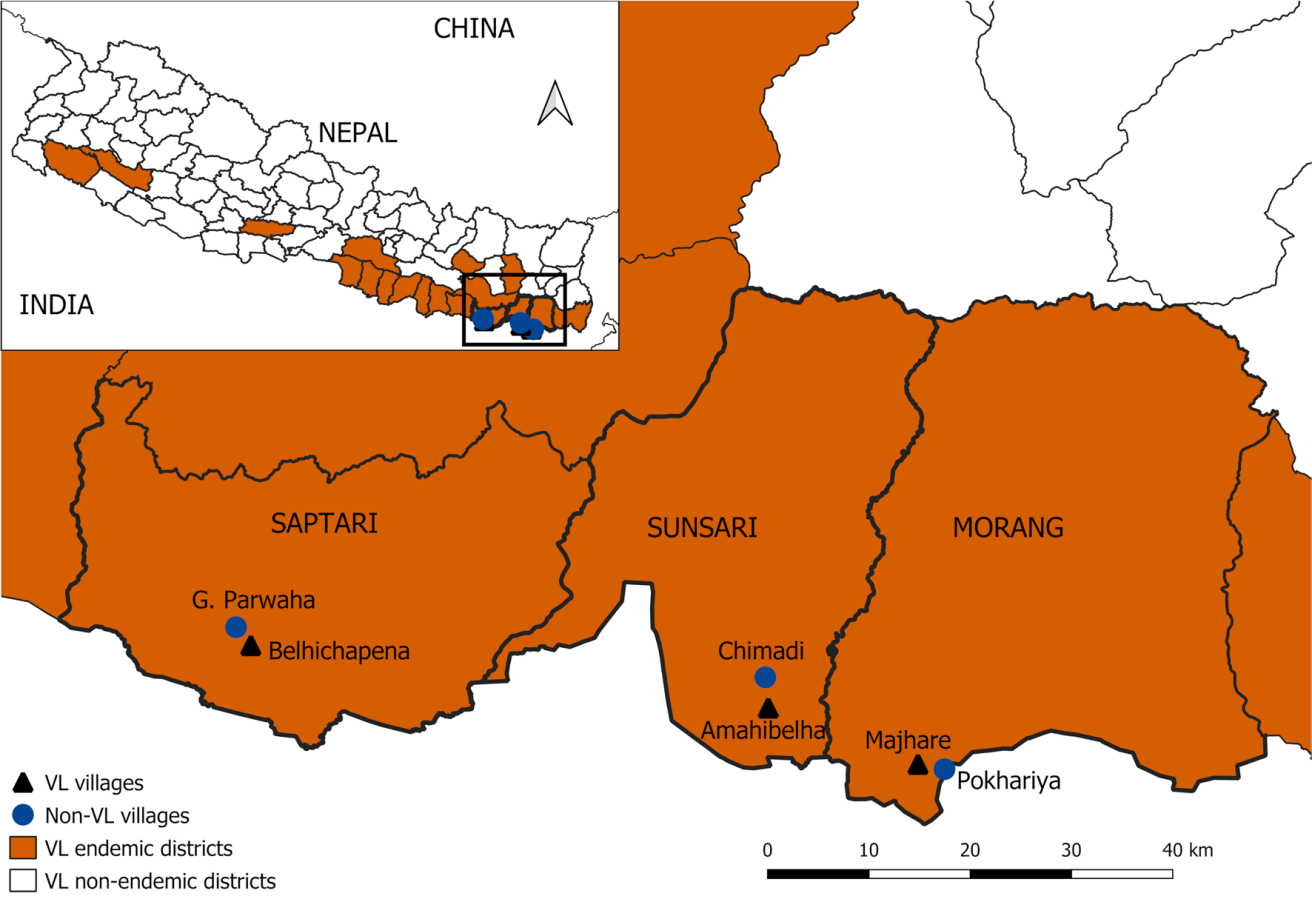
Map of Nepal showing visceral leishmaniasis endemicity (situation as of the year 2016–2017) (inset) and the locations of the study districts and villages. VL villages, villages reporting VL cases in three consecutive years prior to the inception of the study. Non-VL villages, villages without any reported VL cases in the last 10 years before the start of the study. The map was produced with QGIS (version 3.10.2) with open access shapefile. (https://opendatanepal.com/dataset/new-political-and-administrative-boundaries-shapefile-of-nepal)

Three VL endemic districts in eastern Nepal, viz. Morang, Sunsari and Saptari (Fig. 1), were selected for field work. We selected these districts based on (i) the cumulative VL case load over the years 2014–2017 (Additional file 1: Table S1) and (ii) their proximity to the BP Koirala Institute of Health Sciences (BPKIHS), Dharan, Nepal. In each district, one village reporting VL cases in three consecutive years prior to the inception of the study was assigned as the ‘VL village’, and a second village without any reported VL cases in the last 10 years before the start of the study was selected as ‘non-VL village’. Corresponding VL and non-VL villages in each district were carefully selected within 5–10 km distance of each other to minimize the differences in spatial, ecological and housing characteristics. These VL and non-VL villages were Majhare (26.406992 N, 87.337322 E) and Pokhariya (26.401985 N, 87.363547 E) in Morang district, Amahibelha (26.466103 N, 87.188618 E) and Chimadi (26.492791 N, 87.186457 E) in Sunsari district and Belhichapena (26.524450 N, 86.677925 E) and Gamhariya Parwaha (26.541511 N, 86.663169 E) in Saptari district respectively (Fig. 1).

### Sand fly collection and morphological identification

In each of the selected villages, 10 households including human dwellings and mixed dwellings (cattle sheds sharing space with humans) were purposively selected for longitudinal monitoring of the sand fly density for 12 consecutive months, from July 2017 until June 2018. Centre for Disease Control and Prevention miniature light traps (Model 2836 BQ; BioQuip Products, Rancho Dominguez, CA 90220, USA) were used as a standard method of sand fly capturing. One light trap (LT) was placed in the inner lower corner of the main room or cattle shed of each of the selected households and kept functioning for 12 h starting at 18 h to 6 h. In the consecutive morning, resting sand flies were collected using mouth aspirators spending 15 min time in the same household where LT was placed. Both LT and aspirator collections were made for three consecutive nights and mornings in all 10 houses of a village every month during the study period.

We transferred the LT and aspirator collections to the BPKIHS entomology laboratory on a daily basis for the morphological identification of the sand flies using a binocular microscope and regional keys [24, 25]. All sand flies were preserved in 80% ethanol and refrigerated at 4 °C. Male *P. argentipes* and other species of sand flies were stored at the village level per month. Female *P. argentipes* were segregated and stored at the physiological stages (unfed, fed and gravid) at the household level by collection method (light trap versus aspirator) and place of collection (human dwelling and mixed dwellings) level every month.

### Molecular analyses of the *P. argentipes* sand flies

#### DNA extraction

Genomic DNA was extracted from pools of gravid *P. argentipes* sand flies for *Leishmania* detection. Pooling of the gravid *P. argentipes* was done by collection method (LTs and aspirator), household, village and the survey month. Each pool was comprised of a minimum one to a maximum 20 sand flies. In case of more than 20 gravid sand flies collected at an event, the number was divided into two or more pools each containing not more than 20 specimens. Besides, DNA was extracted from individual specimens of blood-fed vector sand flies for blood meal source identification. For reference purpose, DNA extractions were also obtained from blood samples (approximately 5 ml each) of vertebrate hosts like buffalo, cow, goat, pig, rabbit, chicken, duck and pigeon.

The sand flies were crushed using a sterile micro-pestle and homogenized before following the DNA extraction procedure using DNeasy Blood and Tissue Kit (Qiagen, Hilden, Germany) according to the manufacturer’s instructions. DNA elution of 50 µl was collected. The quantity and purity of the DNA extract were checked with a NanoDrop2000 spectrophotometer (Thermo Scientific, DE). DNA extracts were preserved at − 20 °C until being subjected to specific polymerase chain reactions.

#### Detection of *Leishmania* infection in vector sand flies

Assessment of *Leishmania* infection was done on pools of all gravid *P. argentipes* collected throughout the year. A TaqMan probe-based real-time PCR assay targeting the 115 bp length of the small-subunit ribosomal RNA gene of *Leishmania* (SSU-rRNA) was conducted on DNA extracts from the pools of gravid *P. argentipes*. A Leish 18S Set (LE18S) (Integrated DNA Technologies, Belgium) was used in the assay with already described and tested primers: M18S-F-L (5′-CGT AGT TGA ACT GTG GGC TGT GC-3′) and M-18S-R-L (5′-ACT CCC GTG TTT CTT GTT TCT TTG AA-3′) [17, 26], along with a double quenched L-probe (FAM-CTGGTCGTCCCGTCCATGTCGGATT-BHQ1 ZEN). The PCR reaction was carried out in a final volume of 25 µl containing 0.4 µM M18S-F-L, 0.4 µM M-18S-R-L and 0.1 µM L-probe, 1X GoTaq Probe qPCR Master Mix (Promega, USA), 0.1 mg/ml bovine serum albumin (Promega, USA) and 2.5 µl sample DNA. The PCR amplification was conducted in the Rotor-GeneQ real-time PCR System (Qiagen, Hilden, Germany). The thermal profile optimized for the reaction was the activation of the Taq polymerase present in Go Taq Probe Master Mix at 94 °C for 15 min and then 45 cycles of denaturation at 94 °C for 30 s, annealing at 60 °C for 30 s and extension at 72 °C for 45 s followed by the final extension at 72 °C for 5 min. In each PCR run, one positive control consisting of 1 pg of *L. donovani* DNA (BPK282/0) and one negative control containing PCR grade water were included for quality control of the test. Additional qPCR assay targeting the conserved and highly expressed spliced-leader [27] mini-exon sequence [28] was performed to verify the outcomes of the SSU-rRNA qPCR assay.

After the detection of the *Leishmania*-positive pools, *Leishmania* species identification was performed by amplification and sequencing of part of the internal transcribed spacer 1 (ITS-1) region [29, 30]. SSU-rRNA and SL-RNA positive samples were subjected to an ITS-1 nested PCR. A 25 µl PCR reaction mixture contained 1× GoTaq® G2 Master Mix (Promega, USA), 5 µl sample and 0.5 µM forward (5ʹ-CTG GAT CAT TTT CCG ATG-3ʹ) and reverse (5ʹ-TGA TAC CAC TTA TCG CAC TT-3ʹ) primer. Samples were tested on a Bio-Rad T100 thermal cycler using a 5 min activation step at 95 °C, 35 amplification cycles under optimized conditions (30 s at 95 °C, 30 s at 53 °C and 30 s at 72 °C) and a 10 min final elongation at 72 °C. PCR products were checked by 1% agarose gel electrophoresis and purified using a QIAquick PCR Purification Kit (Qiagen, Hilden, Germany) after which they were subjected to a nested PCR reaction using the same conditions as described above. PCR samples were again checked by 1% agarose gel electrophoresis and purified with a QIAquick PCR Purification Kit after which they were sent for Sanger sequencing on an Applied Biosystems 3730XL DNA Analyzer at the Neuromics Support Facility of the University of Antwerp. *Leishmania* species identification was analyzed using SnapGene and NCBI BLAST.

#### Detection of blood meal sources

A targeted ~ 350 bp segment of vertebrate cytochrome *b* (*Cytb*) gene was amplified using the universal primers *Cytb1*-F (L14841: 5′-CCA TCC AAC ATC TCA GCA TGA TGA AA-3′) and *Cytb2*-R (H15149: 5′-GCC CCT CAG AAT GAT ATT TGT CCT CA-3′) [31, 32] for host identification. Each PCR was performed on a 25 μl reaction mixture containing 1× GoTaq Green Master Mix with 2 mM MgCl_2_ (Promega, USA), 0.4 μM each of two primers (Biolegio, The Netherlands) and 5 μl DNA template. Each set of PCR reactions was accompanied by a negative control having PCR grade water and a positive control having DNA extract from human blood. The PCR reaction was performed on an Eppendorf Mastercycler pro PCR System (Hamburg, Germany) setting the thermal profile as pre-activation step at 95 °C for 2 min, followed by 40 cycles of denaturation at 92 °C for 30 s, annealing at 55 °C for 45 s and extension at 72 °C for 1 min and a final extension at 72 °C for 10 min. The PCR products, positive and negative controls were loaded on 2% agarose gel (Eurogentec, Belgium), stained with ethidium bromide (Promega, USA) and examined under a UV trans-illuminator/gel documentation system (Syngene, Cambridge, UK). PCR products (amplicons) were outsourced for Sanger sequencing at the Macrogen Company, South Korea. All generated sequences were checked and edited in BioEdit Sequence Alignment Editor 7.0.5.3 [33] and queried against NCBI BLAST.

#### Molecular validation of *P. argentipes* identification

A subsample of female *P. argentipes* subjected to *Leishmania* infection assessment (*n* = 120) and blood meal analysis (*n* = 31) was verified by DNA barcoding method based on the amplification of targeted 650 bp fragment length of mitochondrial cytochrome c oxidase subunit I (COI) gene as described by Folmer *et al.*, Hebert *et al. *and Kumar *et al*. [34–36].

### Data management and statistical analysis

Entomological data were recorded in a pre-tested paper-based data collection tool and entered in a database developed in Epi Info version 3.5.1 [37]. All statistical analyses and graphical plotting were performed with R software version 4.1.0 [38].

#### Sand fly abundance and seasonality analysis

Sand fly abundance and seasonality analysis were based on the LT collections only, considering them as the standard method of vector collection. Sand flies captured by mouth aspirators were excluded from this analysis to avoid any possible collection bias. Mean number of *P. argentipes* sand flies captured per trap-night per house is represented in a line graph by month, type of village and district. We fitted a generalized linear model (GLM) with a negative binomial distribution to assess the sand fly abundance in function of the type of village, district and month. We used a Bayesian approach to better account for the uncertainty of the estimates using the R package “rstanarm” [39, 40]. This approach produces a 95% credible interval, which means that given the observations, there is a 95% chance that the value lies between the limits of the credible interval [41]. Results of the analysis are represented as an incidence rate ratio (IRR) and credible interval (CI) at 95%.

#### Estimation of the prevalence of *Leishmania* infection rate

For the assessment of *Leishmania* infection, all gravid *P. argentipes* sand flies collected in both LTs and aspirators were used. The prevalence of *Leishmania* infection in pools of unequal size was estimated by a Bayesian Markov Chain Monte Carlo (MCMC) model as described by Speybroeck *et al.* [42, 43]. In this method, prior knowledge of the diagnostic test (in this case, the SSU-rRNA PCR method for detection of *Leishmania* in pools of vector sand flies) characteristics (sensitivity and specificity) was incorporated with the test results. We assumed an imperfect sensitivity, ranging uniformly between 60 and 95%, and a perfect specificity for the PCR test [26]. The result obtained was a posterior probability distribution of the prevalence; the mean and credible intervals at 95% were then calculated from it. Prevalence was estimated using the “truePrevPools” function in the R package “prevalence” [44], and credible intervals (CI) were calculated in the R package “bayestestR” [45, 46].

#### Blood meal analysis

A subsample of 50 blood-fed *P. argentipes* collected in both light traps and aspirators from mixed dwellings of each village during the initial months of the surveys was used for blood meal analysis. Host preference was calculated as the percentage of *P. argentipes* sand flies fed on a specific host relative to the total number of blood-fed *P. argentipes* tested per village category (i.e. VL versus non-VL villages).

## Results

### Sand fly abundance and seasonality

A total of 39,692 *Phlebotomine* sand flies were captured by both CDC light traps and manual aspirators from six villages of three districts during the 12 months of the survey. The vector species *P. argentipes* (*n* = 31,382; 79.1%) was dominant compared to other sand fly species *Phlebotomus papatasi* (*n* = 1632; 4.1%) and *Sergentomyia* spp. (*n* = 6678; 16.8%).

Considering the vector sand flies collected in light traps only (*n* = 20,101), males (*n* = 11,725; 58.3%) outnumbered females (*n* = 8376; 41.7%). About double the number of *P. argentipes* sand flies were captured per trap-night per house in non-VL villages (*n* = 12.66; IRR = 1.90; CI at 95% = 1.66–2.17) compared to VL villages (*n* = 6.13). Distinct variation in vector density was observed at the district level with higher per trap-night per house collections in Saptari (*n* = 15.47; IRR = 1.05; CI at 95% = 0.89–1.23) and lower in Sunsari (*n* = 3.35; IRR = 0.36; CI at 95% = 0.31–0.41) compared to collections from Morang (*n* = 9.35). At district level, vector density per trap-night per house was lower in the non-VL village (*n* = 5.55; IRR = 0.53, CI at 95% = 0.44–0.63) than in the VL village (*n* = 13.12) in Morang, higher in the non-VL village (*n* = 3.79; IRR = 1.53, CI at 95% = 1.28–1.85) than in the VL village (*n* = 2.92) in Sunsari and almost 12 times higher in the non-VL village (*n* = 28.69; IRR = 12.7, CI at 95% = 10.31–15.76) than in the VL village (*n* = 2.41) in Saptari (Table 1).

**Table 1:** Association of the *Phlebotomus argentipes* sand fly density in VL and non-VL villages in aggregate (combining all three districts), at the individual district level and in monthly collections and their 95% credible intervals

Explanatory variables	Incidence rate ratio			
	Aggregate	Morang	Sunsari	Saptari
Intercept	17.98 (14.82–22.01)	28.28 (22.06–36.94)	3.60 (2.69–4.82)	5.25 (3.82–7.49)
Village type (ref: VL Village)				
Non-VL Village	1.90 (1.66–2.17)	0.53 (0.44–0.63)	1.53 (1.28–1.85)	12.7 (10.31–15.76)
District (ref: Morang)				
Saptari	1.05 (0.89–1.23)	–	–	–
Sunsari	0.36 (0.31–0.41)	–	–	–
Months (ref: July 2017)				
August 2017	0.51 (0.4–0.66)	0.45 (0.32–0.65)	0.61 (0.41–0.92)	0.49 (0.32–0.75)
September 2017	0.68 (0.53–0.88)	1.03 (0.71–1.48)	0.77 (0.52–1.17)	0.37 (0.24–0.57)
October 2017	0.45 (0.34–0.57)	0.50 (0.35–0.72)	0.77 (0.51–1.15)	0.39 (0.26–0.6)
November 2017	0.57 (0.44–0.73)	0.32 (0.23–0.46)	0.95 (0.63–1.42)	0.66 (0.44–1.01)
December 2017	0.11 (0.08–0.14)	0.12 (0.08–0.17)	0.38 (0.26–0.59)	0.04 (0.02–0.06)
January 2018	0.00 (0.00–0.00)	0.00 (0.00–0.00)	0.01 (0.00–0.03)	0.00 (0.00–0.00)
February 2018	0.00 (0.00–0.01)	0.00 (0.00–0.01)	0.01 (0.00–0.03)	0.00 (0.00–0.00)
March 2018	0.25 (0.20–0.32)	0.33 (0.23–0.48)	0.52 (0.34–0.78)	0.60 (0.38–0.92)
April 2018	0.34 (0.27–0.45)	0.47 (0.33–0.67)	0.90 (0.62–1.36)	0.15 (0.10–0.24)
May 2018	0.46 (0.35–0.59)	0.22 (0.15–0.32)	1.23 (0.84–1.83)	0.84 (0.54–1.27)
June 2018	0.83 (0.64–1.07)	0.70 (0.49–1.00)	1.90 (1.28–2.84)	0.85 (0.55–1.29)

**Fig. 2:**
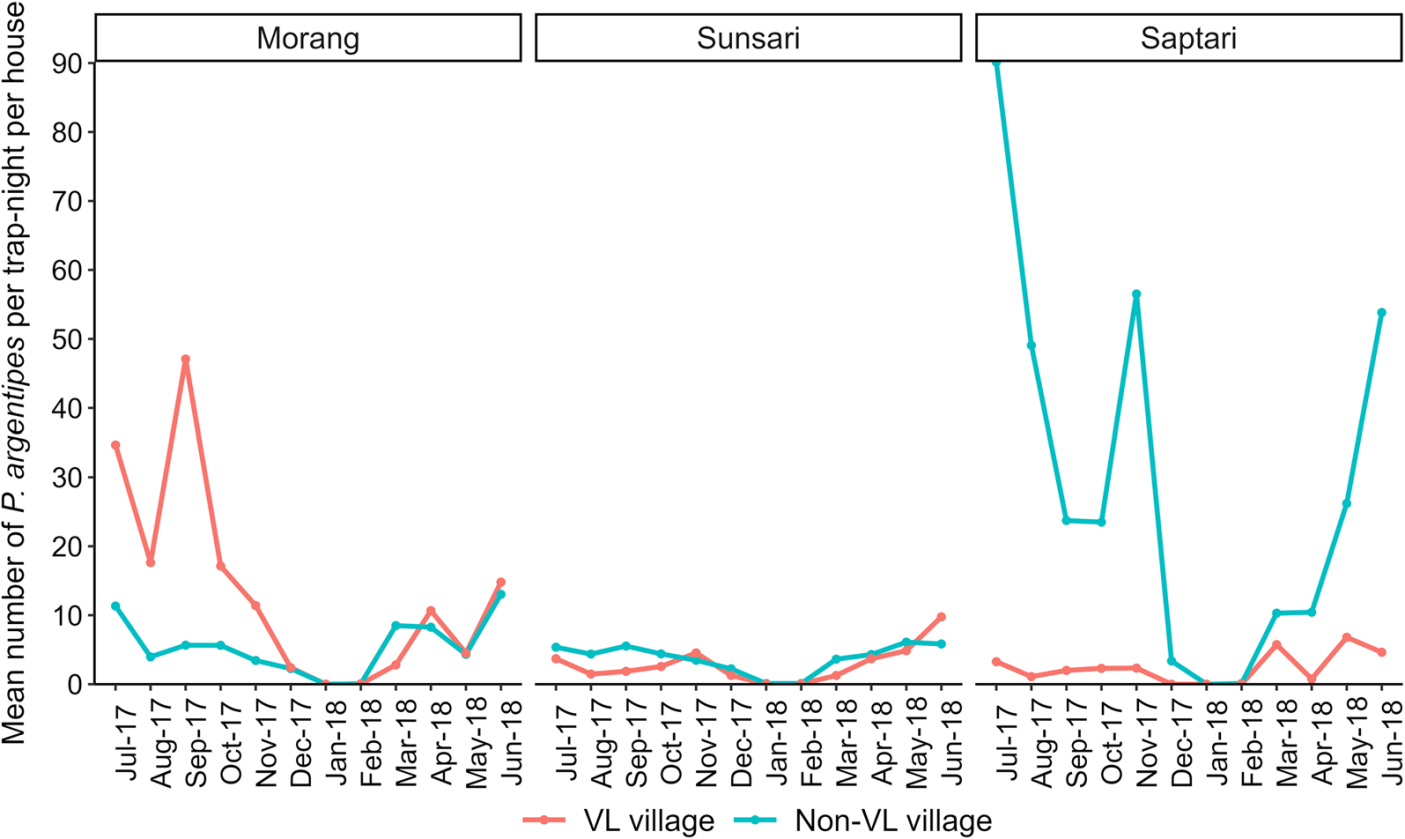
Monthly distribution and seasonality of *Phlebotomus argentipes* in light trap collections in Morang, Sunsari and Saptari districts

Month-wise abundance and seasonality of vector sand flies varied at different degrees in all three districts (Table 1, Fig. 2). In Morang, peaks in sand fly density were seen in July and September 2017 in the VL village and in July 2017 and June 2018 in the non-VL village (Table 1, Fig. 2). In Sunsari, peaks in sand fly density were observed during November 2017 and June 2018 in the VL village, while density peaks were not very clear in the non-VL village. In Saptari, many *P. argentipes* were captured in the non-VL village compared to all other villages year round, and the highest density peaks were observed in the months of July and November in 2017 and June in 2018. In the VL village of the same district, density peaks were seen in the months of March and May 2018. Finally, in the analysis based on the cumulative collections from all the villages, peaks in *P. argentipes* density were marked in the months of June and July and from September to November while the density dropped to almost zero in the months of January and February (Fig. 2, Additional file 2: Table S2).

### Prevalence of *Leishmania* infection in vector sand flies

*Leishmania* infection was assessed in 796 pools of 3735 gravid *P. argentipes* sand flies in total. *Leishmania* infection was detected in four (1.37%) pools out of 293 from VL villages and one (0.2%) pool out of 503 from non-VL villages. The estimated prevalence of *Leishmania* infection in vector sand flies was 2.2% (CI at 95% = 1.1%–3.7%) in VL villages and 0.6% (CI at 95% = 0.2%–1.3%) in non-VL villages (Table 2). These *Leishmania*-infected vector sand flies were collected in the months of May, September and November. Three of five SSU-rRNA-positive pools were confirmed using the SL-RNA PCR. The *Leishmania* species was identified and confirmed as *L. donovani* in two pools from VL villages and one pool from a non-VL village. ITS-1 nested PCR was unsuccessful in two positive pools from a VL village (Table 3).

**Table 2:** Overview of the number of gravid *Phlebotomus argentipes* tested, *Leishmania*-positive pools and estimated infection rate with 95% credible intervals in VL and non-VL villages

Type of village	District	Village	No. of pools	No. of sand flies	No. of positive pools	No. of sand flies in positive pools	Month of collection (positive pools)	Estimated *Leishmania* infection rate in % (95% CI)
VL villages	Morang	Majhare	128	485	0	0		2.2 (1.1–3.7)
	Sunsari	Amahibelha	88	281	1^*^	1	Nov 2017	
	Saptari	Belhichapena	77	301	3^*,†^	7	Sep 2017, Nov 2017, May 2018	
Sub-total			293	1067	4	8		
Non-VL villages	Morang	Pokhariya	133	533	0	0		0.6 (0.2–1.3)
	Sunsari	Chimadi	141	466	0	0		
	Saptari	G. Parwaha	229	1669	1	20	Nov 2017	
Sub-total			503	2668	1	20		
Total			796	3735	5	28		1.1 (0.6–1.8)

^*^Sand flies collected from the adjoining house of a VL patient

^†^Sand flies collected from past VL patient’s house

**Table 3:** *Leishmania* species identification based on ITS-1 sequencing in *Leishmania*-specific SSU-rRNA-positive samples

Sample ID	Type of village	Village	*Leishmania* species	NCBI accession number	Identities	Identities (%)	Remarks
209	VL	Belhichapena	Negative	–	–	–	SSU-rRNA positive/SL-RNA negative
400	VL	Belhichapena	*L. donovani*	MT548853.1	316/316	100	SSU-rRNA positive/SL-RNA positive
431	VL	Amahibelha	*L. donovani*	MT548853.1	316/316	100	SSU-rRNA positive/SL-RNA positive
449	VL	Belhichapena	Negative	–	–	–	SSU-rRNA positive/SL-RNA positive
474	Non-VL	G. Parwaha	*L. donovani*	MT548853.1	316/316	100	SSU-rRNA positive/SL-RNA negative

### Blood meal sources and host preference patterns

**Fig. 3:**
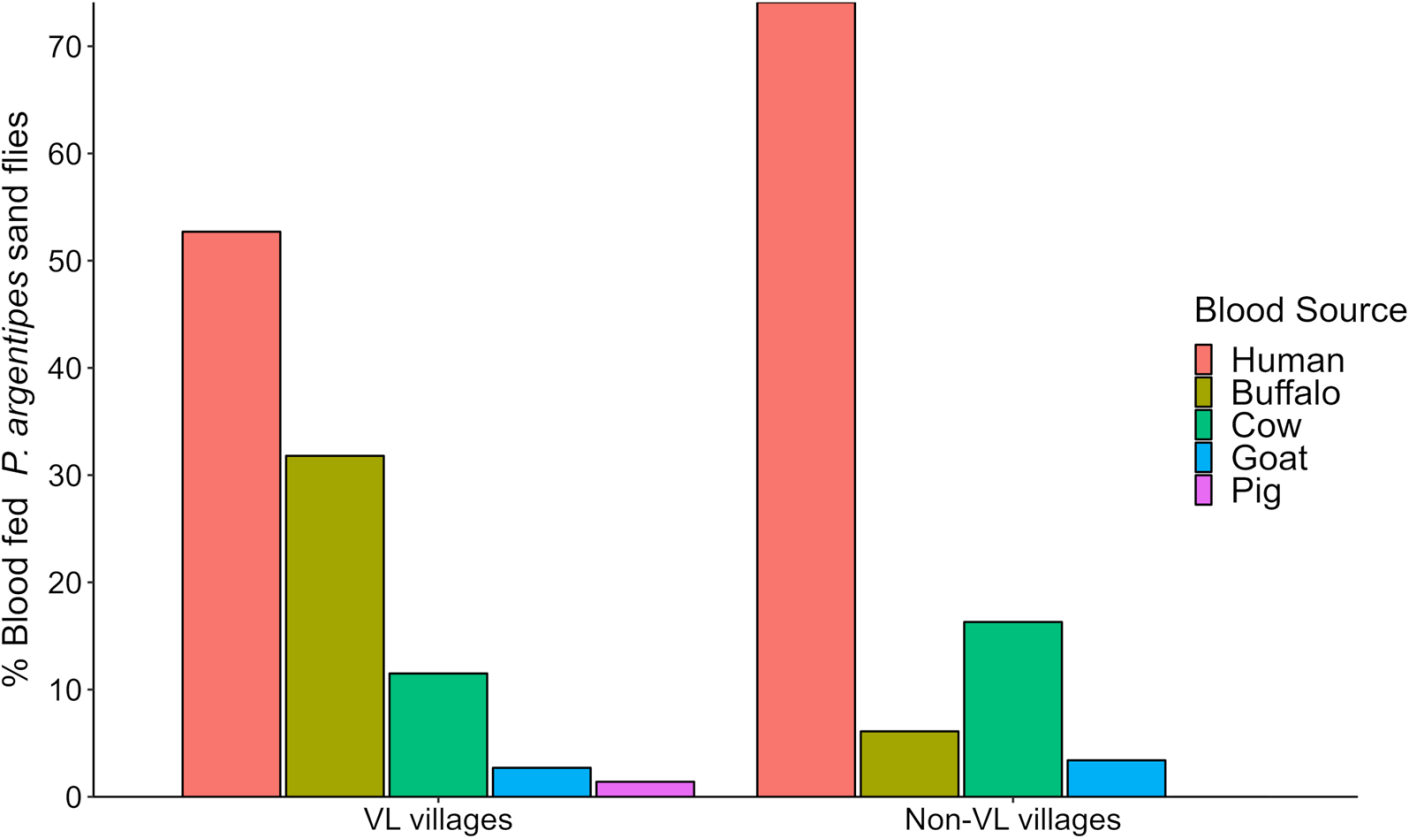
Blood meal sources of *P. argentipes* sand flies captured from mixed dwellings in VL and non-VL villages

A sample of 301 of 3443 fed *P. argentipes* collected from mixed dwellings in all six villages was assessed for identification of the source of blood meal, and vertebrate host species were successfully identified in 295. We submitted selected sequences of the *cytb* gene of the host species to GenBank (accession nos. OQ535519–OQ535565). The most common sources of the blood meal were humans (63.4%; 187/295), followed by domestic buffaloes (18.9%; 56/295), cows (13.9%; 41/295), goats (3.1%; 9/295) and pigs (0.7%; 2/295). In both categories of villages, *P. argentipes* fed primarily on humans (52.7%; 78/148 in VL vs. 74.2%; 109/147 in non-VL villages). Buffaloes (31.8%; 47/148) in VL villages and cows (16.3%; 24/147) in non-VL villages were the second preferred hosts over goats (2.7%; 4/148 in VL vs. 3.4%, 5/147 in non-VL villages). A blood meal from pigs was present in only two fed *P. argentipes* from VL villages (Fig. 3).

### Molecular validation of *P. argentipes* species

The samples of morphologically identified *P. argentipes* sand flies subjected to the assessment of *L. donovani* infection and blood meal analysis were confirmed as the same species by DNA barcoding method.

## Discussion

*Phlebotomus argentipes* sand fly density varied among districts and village types. In one of the districts, *P. argentipes* density was higher in the VL village than in the non-VL village, the other districts had higher collections in non-VL villages. The *P. argentipes* density fluctuated with the season with an increase in June and July and again in September to November. Interestingly,* L. donovani*-infected vector sand flies were present in both VL and non-VL villages. Furthermore, *P. argentipes* showed a high affinity for human blood in mixed dwellings in both types of villages.

Information on seasonality of vector sand flies is crucial to identify the peak season of human-vector contact and thus the period of maximum risk of *L. donovani* transmission. Based on the previous studies and information on seasonality [13, 47, 48], the national VL elimination program in Nepal has been following the schedule of two rounds of IRS using synthetic pyrethroids (alphacypermethrin, deltamethrin or lambda-cyhalothrin) in the reported VL areas or villages, the first round in the months of May–June and second in September–October [6]. The observed seasonality of vector sand fly density in this study was similar to other studies conducted in Nepal [13, 47] and bordering areas of India [49–51] and aligns with the existing practice of IRS. Nevertheless, the seasonality of vector abundance is conducive for *Leishmania* transmission throughout the year except in the months of January and February and raises concerns about the suitability of two rounds of IRS with pyrethroids, the insecticide group that is reported effective to reduce the vector sand fly density up to 4 weeks only in Nepal and Bangladesh [12, 27, 52, 53]. These findings argue for the re-evaluation of vector control strategy in terms of the number of IRS rounds to be considered to cover the long transmission season or to switch or adapt to alternative insecticides that have proven longer residual impact on the sand fly density.

The *Leishmania* infection in vector sand flies as another key indicator of ongoing transmission was also assessed in this study. Some of the previous studies conducted in Nepal and neighboring parts of India during the earlier years of VL elimination showed a range of 0.5% to 17.4% prevalence of *Leishmania* infection in vector sand flies [15–17, 54–56]. In these investigations, prevalence of infection, however, was supposedly influenced by the type of PCR test used, the statistical method to estimate infection rate and the physiological stages of the female *P. argentipes* (unfed, fed and gravid). Thus, the selection of only gravid *P. argentipes* for the *Leishmania* infection assessment in this study was to ensure the sand flies had taken a blood meal at least once and that the meal had already been digested. This way, we assumed the sand flies positive for *Leishmania* to be a proxy for infective sand flies and thus able to transmit the parasite during their next blood meal. Two PCR techniques were used for quality assurance and molecular identification confirmed the *L. donovani* species in the infected vector sand flies. All four *L. donovani*-infected pools from VL villages originated from either the house of an ex-VL patient or directly adjoining houses of the same. Interestingly, we detected an *L. donovani*-positive pool from a non-VL village as well, supporting the findings of the existence of asymptomatic infection in the human population in the areas with zero reported VL cases for more than a decade prior to the study [57]. The presence of infected vector sand flies even in the so-called non-VL village indicates the circulating *Leishmania* parasite in vector and human populations and could possibly cause resurgence of cases in these areas if left unnoticed or neglected. Thus, it is anticipated the program needs to remain vigilant for the existence of transmission even at times when VL case reporting from the longstanding endemic districts is scanty or null.

We found that vector sand flies collected from mixed dwellings had a high affinity towards humans. In mixed dwellings, sand flies were assumed to have equal access to the various types of hosts and minimal influence of biotopes on the feeding behavior. The high human blood index reported in previous studies from Nepal [21, 22] well supports the findings of our study. Despite *P. argentipes* being marked as an opportunistic feeder, many of the recent studies reported a high human blood index in collections from various biotopes including mixed dwellings [19, 50]. From our observations, we do not have evidence of a substantial change in feeding behavior (i.e. anthropophilic to zoophilic) of *P. argentipes* even after years of IRS in endemic districts; hence, the species still possesses a high potential for parasite transmission in the domestic environment.

The main strength of this study is that we assessed the risk of ongoing *L. donovani* transmission in VL endemic districts during the elimination era based on multiple entomological indicators. These indicators were well documented from two contrasting VL epidemiological settings, i.e. VL and VL non-villages within endemic districts, which, to our best knowledge, is the first study of this kind in Nepal. This study is also the first in 15 years to update our knowledge on seasonality, prevalence of *Leishmania* infection and host preference of the vector sand flies in endemic districts of Nepal. However, this entomological evidence would have been complemented by epidemiological and serological data for a complete situation analysis of *L. donovani* transmission in these VL endemic areas where the caseload has been reduced below the elimination threshold in recent years.

## Conclusions

Our findings highlight the risk of ongoing *L. donovani* transmission in villages with and without reported VL cases within VL endemic districts included in the disease elimination program and that *P. argentipes* is still an efficient vector. The favorable entomological indicators for *Leishmania* transmission in non-VL villages emphasize the remaining threats to the VL elimination program and show that it needs to remain vigilant for the re-appearance of transmission even years after the last VL case has been reported from the endemic districts. In addition, we recommend that the program should strengthen the entomological and epidemiological *Leishmania* surveillance in Nepal to better adapt the disease elimination strategies. Also, we recommend conducting similar studies in other VL endemic districts representing various geo-ecological zones and administrative regions to monitor *Leishmania* transmission and track the progress towards elimination.

### Supplementary Information

**Additional file 1: Table S1.** The cumulative caseload in VL endemic and non-endemic districts in the past 3 years, 2014–16, prior to the inception of the sand fly collection.

**Additional file 2: Table S2**: The total number of *Phlebotomus argentipes* collected by CDC light traps by district, type of village and month (30 trap-nights per village per month).

## Data Availability

All datasets generated and analyzed during the study are included in the manuscript. The generated sequences for blood meal identification are submitted in GenBank under accession nos. OQ535519–OQ535565.
