# Supplementary material for: The ongoing risk of Leishmania donovani transmission in eastern Nepal: an entomological investigation during the elimination era
Source: Parasit Vectors. 2023 Nov 6;16:404. doi: 10.1186/s13071-023-05986-9 (PMC10629032; doi:10.1186/s13071-023-05986-9)
Supplement: Supplementary file 1 — Additional file 1: Table S1. The cumulative caseload in VL endemic and non-endemic districts in the past 3 years, 2014–16, prior to the inception of the sand fly collection. [file 13071_2023_5986_MOESM1_ESM.docx]

Additional file 1: Table 1. The cumulative caseload in VL endemic and non-endemic districts in the past three years 2014-16, prior to the inception of the sand fly collection

| S.N | Name of Districts | No. of Cases (2014) | No. of Cases (2015) | No. of Cases (2016) | Endemic/Non-endemic Districts |
| --- | --- | --- | --- | --- | --- |
| 1 | Achham | 0 | 0 | 1 | Non-endemic |
| 2 | Arghakhanchi | 0 | 1 | 2 | Non-endemic |
| 3 | Baitadi | 0 | 0 | 1 | Non-endemic |
| 4 | Bajhang | 0 | 0 | 1 | Non-endemic |
| 5 | Bajura | 0 | 1 | 3 | Non-endemic |
| 6 | Banke | 0 | 1 | 7 | Non-endemic |
| 7 | Bara | 1 | 1 | 1 | Endemic |
| 8 | Bardiya | 0 | 2 | 8 | Non-endemic |
| 9 | Bhojpur | 0 | 5 | 3 | Non-endemic |
| 10 | Bhaktapur | 0 | 0 | 0 | Non-endemic |
| 11 | Dailekh | 0 | 2 | 0 | Non-endemic |
| 12 | Dang | 0 | 1 | 3 | Non-endemic |
| 13 | Darchula | 0 | 2 | 1 | Non-endemic |
| 14 | Dhading | 0 | 0 | 1 | Non-endemic |
| 15 | Dhankutta | 0 | 0 | 2 | Non-endemic |
| 16 | Dhanusha | 10 | 12 | 17 | Endemic |
| 17 | Dolpa | 0 | 1 | 0 | Non-endemic |
| 18 | Doti | 0 | 5 | 2 | Non-endemic |
| 19 | Gulmi | 0 | 1 | 3 | Non-endemic |
| 20 | Humla | 0 | 0 | 3 | Non-endemic |
| 21 | Ilam | 0 | 1 | 2 | Non-endemic |
| 22 | Jajarkot | 0 | 1 | 0 | Non-endemic |
| 23 | Jhapa | 0 | 4 | 12 | Endemic |
| 24 | Kailali | 0 | 4 | 4 | Non-endemic |
| 25 | Kalikot | 0 | 1 | 0 | Non-endemic |
| 26 | Kanchanpur | 0 | 1 | 4 | Non-endemic |
| 27 | Kapilbastu | 0 | 0 | 1 | Non-endemic |
| 28 | Kaski | 0 | 1 | 0 | Non-endemic |
| 29 | Kathmandu | 0 | 2 | 1 | Non-endemic |
| 30 | Kavre | 0 | 0 | 1 | Non-endemic |
| 31 | Khotang | 0 | 0 | 2 | Non-endemic |
| 32 | Lalitpur | 0 | 0 | 0 | Non-endemic |
| 33 | Mahottari | 23 | 30 | 16 | Endemic |
| 34 | Makawanpur | 0 | 1 | 1 | Non-endemic |
| 35 | Morang | 117 | 49 | 53 | Endemic |
| 36 | Mugu | 0 | 0 | 0 | Non-endemic |
| 37 | Nawalparasi | 0 | 0 | 1 | Non-endemic |
| 38 | Nuwakot | 0 | 2 | 0 | Non-endemic |
| 39 | Okhaldhunga | 0 | 1 | 1 | Non-endemic |
| 40 | Palpa | 0 | 6 | 13 | Non-endemic |
| 41 | Parsa | 0 | 0 | 1 | Endemic |
| 42 | Pyuthan | 0 | 2 | 5 | Non-endemic |
| 43 | Ramechap | 0 | 1 | 0 | Non-endemic |
| 44 | Rautahat | 1 | 0 | 0 | Endemic |
| 45 | Rukum | 0 | 0 | 0 | Non-endemic |
| 46 | Rupandehi | 0 | 0 | 2 | Non-endemic |
| 47 | Rolpa | 0 | 2 | 0 | Non-endemic |
| 48 | Rukum | 0 | 1 | 0 | Non-endemic |
| 49 | Salyan | 0 | 1 | 3 | Non-endemic |
| 50 | Sankhuwasava | 0 | 1 | 0 | Non-endemic |
| 51 | Saptari | 27 | 16 | 10 | Endemic |
| 52 | Sarlahi | 24 | 7 | 10 | Endemic |
| 53 | Sindhuli | 0 | 0 | 1 | Non-endemic |
| 54 | Sindhupalchok | 0 | 1 | 0 | Non-endemic |
| 55 | Siraha | 0 | 22 | 22 | Endemic |
| 56 | Sunsari | 14 | 20 | 8 | Endemic |
| 57 | Surkhet | 0 | 6 | 4 | Non-endemic |
| 58 | Syangja | 0 | 3 | 3 | Non-endemic |
| 59 | Tanahu | 0 | 0 | 1 | Non-endemic |
| 60 | Udayapur | 0 | 2 | 2 | Endemic |
|  | Total | 217 | 224 | 243 |  |

(Source: Number of kala-azar cases reported by Districts in the years 2014, 2015, 2016 and 2017. Kala-azar Elimination Programme, Epidemiology and Disease Control Division, Teku, Kathmandu, Nepal)
