# Supplementary material for: The ongoing risk of Leishmania donovani transmission in eastern Nepal: an entomological investigation during the elimination era
Source: Parasit Vectors. 2023 Nov 6;16:404. doi: 10.1186/s13071-023-05986-9 (PMC10629032; doi:10.1186/s13071-023-05986-9)
Supplement: Supplementary file 2 — Additional file 2: Table S2: The total number of Phlebotomus argentipes collected by CDC light traps by district, type of village and month (30 trap-nights per village per month). [file 13071_2023_5986_MOESM2_ESM.docx]

Table S2: The total number of *P. argentipes* collected by CDC light traps by district, type of village, and month (30 trap-nights per village per month)

|  | **Morang** |  | **Sunsari** |  | **Saptari** |  | Sub Total |  | Grand Total |
| --- | --- | --- | --- | --- | --- | --- | --- | --- | --- |
|  | VL village | Non-VL village | VL village | Non-VL village | VL village | Non-VL village | VL villages | Non-VL villages |  |
| July 2017 | 1,040 | 340 | 111 | 161 | 98 | 2,704 | 1,249 | 3,205 | 4,454 |
| August 2017 | 529 | 119 | 44 | 131 | 33 | 1,325 | 606 | 1,575 | 2,181 |
| September 2017 | 1,177 | 147 | 56 | 166 | 60 | 712 | 1,293 | 1,025 | 2,318 |
| October 2017 | 514 | 169 | 77 | 132 | 69 | 705 | 660 | 1006 | 1,666 |
| November 2017 | 342 | 103 | 136 | 104 | 70 | 1,639 | 548 | 1846 | 2,394 |
| December 2017 | 72 | 68 | 37 | 67 | 0 | 101 | 109 | 236 | 345 |
| January 2018 | 0 | 0 | 0 | 3 | 0 | 0 | 0 | 3 | 3 |
| February 2018 | 1 | 2 | 1 | 3 | 0 | 3 | 2 | 8 | 10 |
| March 2018 | 84 | 255 | 38 | 109 | 172 | 309 | 294 | 673 | 967 |
| April 2018 | 320 | 248 | 107 | 129 | 23 | 313 | 450 | 690 | 1,140 |
| May 2018 | 135 | 117 | 141 | 183 | 204 | 786 | 480 | 1086 | 1,566 |
| June 2018 | 444 | 391 | 293 | 175 | 139 | 1,615 | 876 | 2181 | 3,057 |
| Total | 4,658 | 1,959 | 1,041 | 1,363 | 868 | 10,212 | 6,567 | 13,534 | 20,101 |
